# A LAMP-based colorimetric assay to expedite field surveillance of the invasive mosquito species *Aedes aegypti* and *Aedes albopictus*

**DOI:** 10.1371/journal.pntd.0008130

**Published:** 2020-03-04

**Authors:** Bixing Huang, Brian L. Montgomery, Rebecca Adamczyk, Gerhard Ehlers, Andrew F. van den Hurk, David Warrilow

**Affiliations:** 1 Public Health Virology Laboratory, Forensic and Scientific Services, Queensland Health, Brisbane, Queensland, Australia; 2 Metro South Public Health Unit, Metro South Hospital and Health Service, Brisbane, Queensland, Australia; 3 Medical Entomology, Tropical Public Health Services, Cairns and Hinterland Hospital and Health Service, Cairns, Queensland, Australia; Faculty of Science, Mahidol University, THAILAND

## Abstract

**Background:**

Yellow fever, dengue, chikungunya and Zika viruses are responsible for considerable morbidity and mortality in humans. *Aedes aegypti* and *Aedes albopictus* are the most important mosquito vectors involved in their transmission. Accurate identification of these species is essential for the implementation of control programs to limit arbovirus transmission, during suspected detections at ports of first entry, to delimit incursions or during presence/absence surveillance programs in regions vulnerable to invasion. We developed and evaluated simple and rapid colorimetric isothermal tests to detect these two mosquito species based on loop-mediated isothermal amplification (LAMP) targeting the ribosomal RNA internal transcribed spacer 1 (ITS1).

**Methodology/Principal findings:**

Samples were prepared by homogenizing and heating at 99 ^o^C for 10 min before an aliquot was added to the LAMP reaction. After 40 min incubation at 65 ^o^C, a colour change indicated a positive result. The tests were 100% sensitive and species-specific, and demonstrated a limit of detection comparable with PCR-based detection (TaqMan chemistry). The LAMP assays were able to detect target species for various life stages tested (adult, 1^st^ instar larva, 4^th^ instar larva and pupa), and body components, such as legs, wings and pupal exuviae. Importantly, the LAMP assays could detect *Ae*. *aegypti* DNA in mosquitoes stored in Biogents Sentinel traps deployed in the field for 14 d. A single 1^st^ instar *Ae*. *aegypti* larva could also be detected in a pool of 1,000 non-target 1^st^ instar *Aedes notoscriptus*, thus expediting processing of ovitrap collections obtained during presence/absence surveys. A simple syringe-sponge protocol facilitated the concentration and collection of larvae from the ovitrap water post-hatch.

**Conclusions/Significance:**

We describe the development of LAMP assays for species identification and demonstrate their direct application for surveillance in different field contexts. The LAMP assays described herein are useful adjuncts to laboratory diagnostic testing or could be employed as standalone tests. Their speed, ease-of-use, low cost and need for minimal equipment and training make the LAMP assays ideal for adoption in low-resource settings without the need to access diagnostic laboratory services.

## Author summary

Two species of mosquitoes, *Aedes aegypti* and *Aedes albopictus*, are important mosquito vectors that transmit yellow fever, dengue, chikungunya and Zika viruses. There are limited vaccine and antiviral options to treat these diseases, and so vector control (chemical or biological) is the main strategy for preventing virus transmission. Active mosquito surveillance employing a range of methods is critical for guiding control programs. The context where surveillance is undertaken includes assessing population dynamics in areas where *Ae*. *aegypti* and *Ae*. *albopictus* are established and transmitting viruses; delimiting their range during incursions; detecting importations at first ports of entry; or conducting broad-scale presence/absence surveillance in regions vulnerable to invasion. Effective surveillance is underpinned by accurate and rapid identification of collected specimens. Molecular methods can complement more traditional morphological identification in instances when sample numbers are large, specimens are damaged, when related species share overlapping morphology or when diagnostic features in early instar larvae are not developed. Laboratory-based DNA tests are available; however, these require sophisticated equipment and incur lag time for the sample to be shipped and tested, which results in critical delay to implement rapid intervention. In this work, we have developed simple rapid tests to detect *Ae*. *aegypti* and *Ae*. *albopictus* that can be performed at or near the field sites, require only a single reaction temperature, and which give a visual colour change when a positive sample is detected. The assays identified all life stages and body components (legs and wings) tested, as well as adult specimens that were held in traps under field conditions for two weeks. They also detected a single 1st instar larva in a sample of >1,000 non-target mosquitoes to expedite ovitrap processing and subsequent morphological identification. The assays should be a useful adjunct to laboratory testing or could be employed as standalone tests.

## Introduction

Arboviruses are arthropod-borne viruses that occur worldwide and cause a large burden of human mortality and morbidity. Some of the most important arboviruses are yellow fever (YFV), dengue (DENV), chikungunya (CHIKV) and Zika (ZIKV) viruses, all of which are transmitted primarily by the invasive and urbanized mosquito species, *Aedes aegypti* and *Aedes albopictus* [1]. *Aedes aegypti* originated in Africa [2]. Its historical dispersal was facilitated by its anthropophilic behaviour, and accelerated by the human movement and trade that accompanied the period during and after European colonialism. Despite successful attempts at eradication in the 20^th^ Century using large-scale insecticide spraying, and removal or remediation of artificial containers that provide larval habitats (source reduction), the vector is still widespread in tropical and sub-tropical regions of the world [3]. By comparison, *Ae*. *albopictus* (the “Asian tiger mosquito”) originated in Southeast Asia and has spread more recently to become an important arbovirus vector in infested regions. It has colonized parts of Africa, North and South America, Europe and the islands of the Western Pacific [4], with much of its dispersal linked to the used tire trade [5]. As well as being an efficient arbovirus vector, the mosquito is an aggressive biter, impacting the quality of life [6, 7]. Statistical models of spatial spread predict that both species are likely to continue to expand their geographical distributions [8].

Except for YFV, effective vaccines for these *Aedes* transmitted arboviruses are not available, and there are no antivirals. Hence, mosquito surveillance and control are currently the most important measures used to reduce virus transmission [9]. Control of *Ae*. *aegypti* and *Ae*. *albopictus* is applied in several different geographical distribution contexts: (a) where the species are established, activities are undertaken to limit virus transmission and pest biting; (b) where they have recently become established, surveys are conducted to delimit their geographical distribution; and (c) in locations free from infestation, but where surveillance is conducted to detect importations or incursions. A number of strategies are available for surveillance of *Ae*. *aegypti* and *Ae*. *albopictus*, ranging from surveys of containers that serve as immature habitats, adult trapping with Biogents Sentinel (BGS) traps or Gravid *Aedes* Traps (GATs), to ovitraps, which collect eggs oviposited by gravid females [10]. The choice of sampling method will depend on the objectives of the surveillance program, geographical and temporal distribution of mosquitoes, logistical and budgetary constraints, and the training of personnel.

Irrespective of the mode of surveillance, molecular methodology can play an integral role in identification of mosquito collections. A number of species-specific PCR-based assays have been developed to identify *Ae*. *aegypti* and *Ae*. *albopictus*, and exist in classical [11] and real-time platforms [12–14]. These assays can be used to differentiate between species which share overlapping morphology, such as *Ae*. *albopictus*, which is difficult to distinguish from the native Australian species, *Ae*. *scutellaris*, in the larval stage [15]. Molecular analyses can also be used to identify field samples damaged during the collection process or pupal exuviae, a scenario faced by quarantine personnel inspecting cargo receptacles at ports of first entry. Finally, molecular identification of 1^st^ instar larvae is a critical part of a novel system to expedite presence/absence surveys for both of these invasive mosquitoes, the Rapid Surveillance for Vector Presence (RSVP) [13]. In this system, thousands of eggs from multiple ovitrap collections are batched in cohorts of up to 5,000 eggs and analysed using TaqMan PCR.

Whilst molecular assays have an important role to play in surveillance programs, they are restricted to use in laboratories housing complex equipment, such as thermocyclers and real-time PCR machines, which require a reliable electrical supply to operate. A simple, cheap, rapid and accurate field test would be a useful tool in identifying mosquito species during front-line surveillance and control activities. Such a test could be used in remote locations, at ports of first entry, and other rudimentary facilities without requiring access to specialized laboratories and equipment. In this study, we demonstrate proof-of-principle of a rapid colorimetric loop-mediated isothermal amplification (LAMP) based field test, which requires only minimal sample preparation. The *Ae*. *aegypti* assay was adapted from [16], whilst we developed the *Ae*. *albopictus* assay as part of the current study. Importantly, we assess applicability of these assays for identification of invasive mosquitoes in different scenarios typically encountered in the field: (a) different life stages or body components; (b) specimens stored in BGS traps for extended periods in the field; and (c) potential detection of low numbers of target species in a background of non-target species in regions vulnerable to invasion, as encountered in the RSVP program.

## Materials and methods

### Ethics statement

The biological material in this work were collected as part of routine public health surveillance activities.

### Mosquito sample preparation

Initial assay development was undertaken using 4^th^ instar larvae of *Ae*. *aegypti* and *Ae*. *albopictus* from colonies which had originated from Innisfail and Hammond Island, north Queensland, Australia, respectively. For the preparation of column-purified mosquito DNA, individual mosquitoes were placed in a 1.5 ml microfuge tube in 0.5 ml of either sterile water or 10 mM Tris pH 8.0 and homogenized with a polyethylene pestle. The homogenate was clarified by centrifugation for 1 min at 2000 X *g*. Nucleic acids were then extracted using the QIAamp Viral RNA extraction kit according to the manufacturer’s instructions (Qiagen, Germany). For the rapid sample preparation, a previous publication had successfully used a heat-based (99 ^o^C for 10 min) treatment to prepare DNA for LAMP [17], and this formed the basis of a modified sample preparation method. The homogenized sample was heated for 10 min at 99 ^o^C and then clarified by centrifugation as described above. The supernatant was transferred to a fresh tube and used immediately or placed at -80 ^o^C for long-term storage. Column purification was used when performing real-time PCR assays. The rapid preparation method was used for LAMP assays with the exception of initial limit-of-detection determination and species specificity when column extractions were used.

### Colorimetric LAMP assay

Primers for the detection of *Ae*. *aegypti* (Table 1) were targeted to the ITS1 ribosomal spacer region [16]. Primers for the detection of *Ae albopictus* were designed targeting the ITS1 region using previously described LAMP assay parameters [18]. To ensure specificity, an *in silico* analysis of the primers was performed on 35 *Aedes albopictus* GenBank entries from around the world, and minimal nucleotide variation was found. The LAMP assay was performed in 0.2 ml clear tubes containing 1x WarmStart Colorimetric Master Mix (New England Biolabs); BIP and FIP primers, 1.6 μM each; B3 and F3 primers, 0.2 μM each; and Loop B and F primers, 0.4 μM each; sample extract (6 μL or sterile Milli-Q water control) in a final reaction volume of 25 μL. The reaction was incubated in a thermal cycler (Applied Biosystems 9700) for 40 min at 65 ^o^C and the reaction terminated using heat inactivation at 80 ^o^C for 10 min. A no-template control (NTC) was always included. If reaction products were generated, the pH indicator phenol red in the reaction mixture changed from purple to yellow. A sample was deemed positive when its corresponding LAMP reaction was yellow in colour and the accompanying NTC reaction was negative (purple colour). A fluorescent probe PCR-based assay (TaqMan) was used for sensitivity comparison [13].

**Table 1:** Primers used in this study.

Target	Primer	Sequence	Reference
*Ae*. *albopictus* ITS1	AlbF3	CCCGACAAGGCAATATGTCG	This study
	AlbFL	GCGGCAGCGTCCTCGGAA	“
	AlbFIP	GTTCCCTCCGATCAGCGAACTCAGGCATGGCCAACCTCTAGC	“
	AlbBL	TACGATACGGCTTGCACAGA	“
	AlbB3	ACCAAAGCGGACGTATGTGC	“
	AlbBIP	AGCTGCGCAATGTCCGTACGCGGCGGGTTCGGCCCTGCTGA	“
*Ae*. *aegypti* ITS1	F3	CAAGGCACGTTACCMGG	[16]
	B3	GCAGCACAACCACACGG	“
	FIP	GAGTGTCGCCGAGAGAAGCCGAGGACACTGCTGCACC	“
	BIP	TCGGACGCTCGTACGTACCGTCGAGCTTCGRCGASACA	“
	FL	TGCGCGTGCCTCCGGGTGA	“
	BL	CGAACGTGTCTGGCGTGTTCTG	“

### Determination of the limit-of-detection, specificity and sensitivity of the assays

For limit-of-detection, extracts were prepared of two mosquitoes of each species, and these were subjected to 10-fold serial dilution. The respective LAMP assay was performed on each sample, and real-time PCR in parallel for comparison. The limit was determined when both dilutions were positive. Sensitivity and specificity testing of the LAMP assays was undertaken using several Australian container-inhabiting mosquito species which had been sourced from colonies, archival material or directly from the field (Table 2). To prevent any possibility of bias in interpretation of the results, samples were placed in random order and blinded to the individual performing the assay.

**Table 2:** Mosquito specimens collected from various locations in Queensland and used for testing of *Aedes aegypti* and *Ae*. *albopictus* LAMP assays.

				LAMP assay tested			
				*Ae*. *aegypti*		*Ae*. *albopictus*	
Species	Origin	Sample type	Number tested	Number (%) positive		% positive	
*Ae*. *aegypti*	Cairns	Adult (F_3_ colony)	5	5	(100)	0	(0)
	Innisfail	4^th^ instar larva (F_5_ colony)	21	21	(100)	0	(0)
		1^st^ instar larva (F_5_ colony)	4	4	(100)	0	(0)
		Leg (from F_5_ colony adult)	5	5	(100)	0	(0)
		Wing (from F_5_ colony adult)	4	4	(100)	0	(0)
		Pupal exuvia (from F_5_ colony pupa)	5	5	(100)	0	(0)
	Townsville	4^th^ instar larva (F_6_ colony)	15	15	(100)	0	(0)
	Cairns	4^th^ instar larva (*w*Mel *Wolbachia* infected)	9	9	(100)	0	(0)
*Ae*. *albopictus*	Hammond Island	4^th^ instar larva (colony, unknown generation number)	43	0	(0)	43	(100)
		1^st^ instar larva (colony, unknown generation number)	4	0	(0)	4	(100)
	Thursday Island	Adult (F_5_ colony)	2	0	(0)	2	(100)
		4^th^ instar larva (F_5_ colony)	7	0	(0)	7	(100)
		Leg (from F_5_ colony adult)	4	0	(0)	4	(100)
		Wing (from F_5_ colony adult)	1	0	(0)	1	(100)
*Ae*. *notoscriptus*	Brisbane	4^th^ instar larva (field collected)	36	0	(0)	0	(0)
		Leg (from field-collected adult)	2	0	(0)	0	(0)
		Wing (from field-collected adult)	1	0	(0)	0	(0)
*Ae*. *scutellaris*	Northern Peninsula Area	Adult (field collected)	6	0	(0)	0	(0)
*Ae*. *tremulus*	Northern Peninsula Area	Adult (field collected)	3	0	(0)	0	(0)
*Ae*. *palmarum*	Cairns	Adult (field collected)	3	0	(0)	0	(0)
*Cx*. *quinquefasciatus*	Brisbane	4^th^ instar larva (field collected)	5	0	(0)	0	(0)

### Identification of life stages and body components

The applicability of the LAMP assays for identification of 1^st^ instar larvae, 4^th^ instar larvae, pupae and adults of *Ae*. *aegypti* and *Ae*. *albopictus* was tested. The ability for the LAMP assays to identify different body components, such as individual legs, wings and pupal exuviae, was also assessed.

### Assessment of LAMP assays for identification of *Ae. aegypti* collected in BGS traps

The ability for the LAMP assays to identify *Ae*. *aegypti* collected in BGS traps deployed for 14 days in Brisbane between March 18 and April 1, 2019 was assessed. Three BGS traps were assembled according to manufacturer’s instructions (Biogents, Regensburg, Germany) and placed in a sheltered location. Three- to four-day old F_5_ Innisfail strain *Ae*. *aegypti* females were killed by freezing at -20 ^o^C for 1 h. Fifteen individual mosquitoes, designated as day 0 samples, were analysed immediately using the *Ae*. *aegypti*-specific LAMP assay. Twenty-five mosquitoes were concurrently placed in each of the 3 BGS traps. On days 1, 3, 7 and 14, 5 mosquitoes were removed from each trap, their identity verified by microscopic examination, before being analysed using the *Ae*. *aegypti*-specific LAMP assay.

### Development of a rapid method to prepare ovitrap collections for detection of target invasive species against a background of non-target endemic species

Herein, the LAMP assays were evaluated for the identification of target species amongst a background of non-target species. As part of this process, we developed a more rapid and robust method for preparing the eggs for analysis that didn’t require additional equipment or consumables, such as a vacuum pump or FTA cards, which are required for the current RSVP system [13].

Initially, we assessed whether the water from the container the eggs hatched in could be used as a sample type for identification, as an adaptation of environmental DNA (eDNA) testing [19]. Innisfail strain *Ae*. *aegypti* eggs were placed in a 750 mL polyethylene container containing 200 mL of Milli-Q water to which ≈ 10 mg of brain-heart powder was added to deoxygenate the water and stimulate hatching. One millilitre of water from containers with between 3 and 111 larvae was removed on the first 3 days and at day 7 post hatching and analysed using the *Ae*. *aegypti* LAMP assay as described above. In a second experiment, either 1 or 5 larvae that had hatched within 2 h were removed from the hatching water and placed in 200 mL of clean Milli-Q water containing 1 Cichlid Staple pellet (Kyorin Co. Ltd, Himeji, Japan) as a food source. Again, 1 mL samples of water were removed on days 1–3 and 7 post hatching and tested using the *Ae*. *aegypti* LAMP assay.

In the final experiment, we tested whether a simple syringe-sponge system could be used to capture the 1^st^ instar larvae at 24 h post hatching. The apparatus consisted of a 30 mL syringe (Becton Dickinson, Franklin Lakes, NJ) from which the plunger had been removed. A circular piece of 1 cm thick sponge (Edco, Marrickville, Australia) was placed in the bottom of the syringe, which was then placed vertically in a clamp stand. The 200 mL of water containing larvae was agitated and poured into the syringe, where larvae became lodged in the sponge matrix. To ensure all larvae had been removed from the container, a further 25 mL of Milli-Q water was added to the container and poured through the syringe. The plunger was then used to evacuate as much of the water as possible before the sponge was removed with long forceps and placed in a 30 mL vial. The rapid sample preparation of the larvae trapped in sponge consisted of adding 2.5 mL of sterile water to the vial, vortexing briefly, heating at 99 ^o^C for 10 min before 6 μL of liquid was added to the LAMP reaction.

To assess the ability of the syringe-sponge method to prepare ovitrap samples for LAMP analysis, pools of various sizes were produced by placing either 1 or 5 1^st^ instar *Ae*. *aegypti* larvae with differing numbers of 1^st^ instar *Ae*. *notoscriptu*s larvae. Pools containing only 1^st^ instar *Ae*. *aegypti* and *Ae*. *notoscriptus* were used as positive and negative controls, respectively.

### Application of the syringe-sponge system for detecting *Ae*. *aegypti* in field-collected ovitrap samples

In the final assessment, field-collected ovitrap samples were obtained from 5 Queensland municipalities: four are located in southeast Queensland (Brisbane, Logan, Redland and Scenic Rim) whereas Cairns is located in north Queensland. *Aedes aegypti* is endemic in Cairns, whilst this species has not been detected in southeast Queensland for over 50 years [13]. Ovitraps were deployed for two weeks, after which egg strips were removed, dried overnight and forwarded to Forensic and Scientific Services, Brisbane, Queensland for analysis. The number of eggs on each ovistrip (material oviposition substrate placed in ovitrap) was enumerated using a proprietary modification of ‘tracking.js’ edge software (copyright Eduardo A. Lundgren Melo 2014, https://trackingjs.com/) that analysed imagery captured through a Logitech C920 webcam (Logitech, Newark, CA) and pools of approximately 1,000 eggs were formed. As described above, the eggs were then hatched, processed using the syringe-sponge system and analysed with the *Ae*. *aegypti* LAMP assay.

## Results

### Development of a rapid colorimetric assay for the detection of *Ae*. *aegypti* and *Ae*. *Albopictus*

LAMP-based assays to both *Ae*. *aegypti* and *Ae*. *albopictus* were designed or adapted to a commercially available isothermal amplification and colorimetric detection system which uses the pH indicator, phenol red, to detect hydrogen ions generated during amplification (S1 Fig). Both tests were targeted to the ITS1 ribosomal spacer region of the respective species. ITS1 has sufficient sequence variation that it enabled the design of species-specific primers. The primers used to detect *Ae*. *albopictus* were designed for this work and the primers used to detect *Ae*. *aegypti* were published previously [16] (Table 1).

To facilitate field application, a rapid and simple sample preparation that avoided the necessity of column or organic solvent-based extraction was used which produced DNA of sufficient purity to enable isothermal amplification. The two assays were compared with previously developed PCR assays for the detection of both species that were based on TaqMan chemistry [13]. When determining the assay’s limit of detection, the highest dilutions of mosquito DNA extracts at which products were detected by LAMP (two mosquitoes each) were 10^5^−10^6^-fold for *Ae*. *aegypti* and 10^4^-fold for *Ae*. *albopictus* (S1 and S2 Tables). This compared with detection by TaqMan assays performed in parallel at threshold cycle numbers (C_t_) of 29–32 for *Ae*. *aegypti* and C_t_ of 30–31 for *Ae*. *albopictus* for the above dilutions, respectively.

To determine their diagnostic performance, both LAMP assays were tested on 68 and 61 samples of *Ae*. *aegypti* and *Ae*. *albopictus*, respectively, and other Australian container-inhabiting species (*Ae*. *katherinensis*, *Ae*. *notoscriptus*, *Ae*. *tremulus*, *Ae*. *scutellaris*, and *Culex quinquefasciatus*) in a blinded test. There was concordance between the species and its respective assay (Table 2). Hence, the sensitivity and specificity for both tests was 100% (*n* = 185).

As well as 4^th^ instar larvae, the assays were able to detect DNA of 1^st^ instar larvae and adults (Table 2). We also showed that the *Ae*. *albopictus* assay was able to detect pupae. Both assays were able to correctly identify species from DNA prepared from pupal exuviae, and legs and wings separated from whole dead mosquitoes.

### Detection of *Ae*. *aegypti* held in BGS traps

The mean maximum and minimum temperature during the BGS trap trial were 30.4 ^o^C and 19.8 ^o^C, respectively; 48.4 mm of rain fell during this time [20]. All *Ae*. *aegypti* could be identified using the *Ae*. *aegypti*-specific LAMP assay, irrespective of the day post-deployment the specimens were removed. Importantly, the LAMP assay did not detect *Ae*. *aegypti* DNA in a single female *Ae*. *notoscriptus* collected and subsequently removed from a trap on day 14.

### Preparation of ovitrap collections for detection of target species within pools of non-target species

Initial attempts to identify *Ae*. *aegypti* in aliquots obtained from the water larvae were hatched in and from water that larvae were transferred into within 2 h post hatch produced variable results (S3 Table). In the first experiment, 100% (6/6) of aliquots removed from hatching water that 3 to 111 larvae had hatched in 24 hours earlier were positive (S3 Table). In the next experiment, only 2 out of 10 (20%) of aliquots taken from water in which 1–5 larvae had hatched in were positive in the LAMP assay at both 24 and 48 h post submersion. In the final experiment, only 5 out of 40 (12.5%) of aliquots removed from water that larvae had been transferred into immediately after hatching were positive; DNA was unable to be detected after 2 d.

Based on the inconsistency of these results, we assessed the newly developed syringe-sponge system for preparation of ovitrap samples. The *Ae*. *aegypti* LAMP assay was able to identify all sample combinations prepared using the syringe-sponge method, including a single 1^st^ instar *Ae*. *aegypti* larva within pools of an estimated 1,000 1^st^ instar *Ae*. *notoscriptus* larvae (Table 3).

**Table 3:** *Aedes aegypti* 1^st^ instar larvae samples prepared using the novel syringe-sponge system and tested with the *Ae*. *aegypti*-specific LAMP assay.

	LAMP result			TaqMan RT-PCR Result				
Species ratio^a^	Number pools tested	Number (%) positive		Number (%) positive		Mean ± SD C_t_ value^b^		
0:100	2	0	(0)	0	(0)	≥ 39		
0:1,000	2	0	(0)	0	(0)	≥ 39		
1:0	4	4	(100)	4	(100)	21.8	±	1.0
1:1,000	5	5	(100)	5	(100)	29.2	±	3.1
5:0	3	3	(100)	3	(100)	21.0	±	0.6
5:95	5	5	(100)	5	(100)	22.5	±	0.7
5:1,000	6	2	(100)	2	(100)	24.6	±	2.4

^a^Ratio of individual *Ae*. *aegypti* to *Ae*. *notoscriptus* in pools of first instar larvae.

^b^A sample was detected if the cycle threshold (C_t_) value was < 39.0 cycles. C_t_ values ≥ 39.0 were considered to be not detected.

### Application of LAMP to identify *Ae*. *aegypti* in field collected ovitrap samples

The syringe-sponge sample preparation system and LAMP assay was assessed on 16 pools formed from 86 ovitrap collections, comprising an estimated 12,369 eggs, obtained from five 5 locations in Queensland (Table 4). *Aedes aegypti* was detected in the 4 Cairns pools, comprising an estimated 816 eggs. In contrast, *Ae*. *aegypti* was not detected in the 12 pools, comprising an estimated 11,553 eggs, processed from southeast Queensland.

**Table 4:** Detection of *Ae*. *aegypti* DNA samples from ovitraps deployed in 5 local councils in Queensland, Australia.

				TaqMan RT-PCR Result		
Collection location^a^	Geographical co-ordinates	Number of ovitraps	Estimated number of eggs collected	Number of pools processed	Number (%) of positive pools	
Brisbane	27^o^30’S, 152^o^58’E	15	3,156	3	0	(0)
Cairns	16°54’S, 145°43’E	23	816	4	4	(100)
Logan	27^o^38’S, 153^o^06’	10	2,565	3	0	(0)
Redland	27^o^31’S, 153^o^15’	31	4,365	4	0	(0)
Scenic Rim	27^o^59’S, 152^o^59’	7	1,467	2	0	(0)

^a^The location refers to the council jurisdiction where ovitraps were deployed.

## Discussion

There are a number of contexts where effective and rapid surveillance of invasive mosquitoes is critical, including during implementation of chemical or biocontrol programs to limit arbovirus transmission, delimiting the extent of incursions, interceptions at ports of first entry and contemporaneous networks of surveillance throughout large urbanized regions that are vulnerable to invasion. Underpinning these activities is accurate identification of collected mosquitoes, which is relatively straightforward to perform with undamaged adult and larval specimens by a trained operator using microscopy but becomes increasingly laborious as sample numbers become extensive. Here, we present rapid and simple assays for the identification of *Ae*. *aegypti* and *Ae*. *albopictus* mosquitoes based on the isothermal LAMP system. We adapted the *Ae*. *aegypti* assay to enable colorimetric product detection based on the phenol red indicator, whilst the *Ae*. *albopictus* colorimetric LAMP assay was designed, developed and validated entirely in this work.

In our hands the LAMP technology was robust and reliable. We have shown that both primer sets work with the colorimetric detection system at levels of sensitivity similar to the real-time TaqMan PCR-based assays described in Montgomery et al. (2017) [13] developed for the identification of the same species of mosquitoes. Importantly, we tested the LAMP assays on the types of samples that have previously been submitted to our laboratories for identification using the real-time TaqMan PCR platform. Considered the optimal trap for collecting adults of both species, the BGS trap can be deployed for short or extended periods in the field. Collected mosquitoes are subjected to high temperature and humidity, and a constant airflow, a combination which has an increasingly deleterious effect on collected mosquitoes over time, damaging key morphological features [21]. We have shown that the LAMP can identify mosquitoes stored for at least 2 weeks in field deployed BGS traps. Furthermore, the ability to detect DNA from only a single leg or wing suggests that even when the rest of the body is destroyed or missing, then they can still be accurately identified using the LAMP assays.

The development of the syringe-sponge sample preparation method and LAMP assays provides the capability for jurisdictions involved in the RSVP program to perform their own molecular diagnostics. These methods could be used to identify samples collected by citizen scientists, who deploy “Do-It-Yourself” ovitraps and submit resultant egg strips for identification during synchronized rounds of trapping in southeast Queensland (https://metrosouth.health.qld.gov.au/zika-mozzie-seeker). This citizen science format is also being trialled in selected schools as a potential focus of their science, technology, engineering and mathematics (STEM) stream. Students could utilize the LAMP assays for sample processing, thus introducing them to molecular techniques.

The attributes of LAMP-based diagnostics remove the requirement for access to a controlled laboratory environment which should enable its application to field testing in local settings in both developed and developing countries. In contrast to PCR-based assays, LAMP assays do not require the purchase of expensive thermocyclers, whilst the use of a brief heat treatment for lysis rather than column or organic solvent-based methods avoid the chemicals and equipment requirement for a full laboratory extraction. A source of constant heat is all that is required. A heating block or water bath can be battery-powered for locations where electrical mains power is not available. Test results are obtained by visual inspection, without the need for gel-based or cell phone visualization [16, 22]. Critically, LAMP assays are cheap, being priced at < $US3.00 per test, which is considerably lower than the ≈ $US20 per test required to extract and perform a real-time TaqMan PCR. This cost efficiency makes possible the much more expansive surveillance programs often not attainable, but required, for informative monitoring of the spatial and temporal heterogeneity of both these and other invasive species.

When applied to a field setting, workflow practices will have to be adopted to minimize template contamination and generation of false positive results. This will necessitate the inclusion of no-template controls, positive control reactions and ensuring that completed reaction tubes are not opened. As WarmStart *Bst* 2.0 DNA polymerase is used for amplification, a positive control template can be added to tubes and then stored prior to usage on-site, further reducing the risk of contamination from positive control material. Access to quality assurance diagnostic tests is recommended to validate positive test results.

In conclusion, the development of LAMP-based testing provides cheap, rapid, specific and sensitive assays for the surveillance and control of mosquito-borne pathogens. The assays may be used as standalone tests, or as an initial screen followed by confirmatory laboratory testing using alternative diagnostic methods under more controlled conditions. Whilst we developed assays for mosquito species identification, LAMP technology has been applied to detection of arboviruses [23] and *Wolbachia pipientis* [22, 24] in *Ae*. *aegypti*. Thus, once LAMP-technology has been adopted for one purpose, it can be adapted to identify other species or serve other applications, with only a change in reagents, particularly primers and probes, being required.

## Supporting information

S1 Fig Examples of the colorimetric *Ae*. *aegypti* and *Ae*. *albopictus* LAMP assays. As indicated, mosquito species were tested in duplicate or triplicate with LAMP reactions to detect (A) *Ae*. *aegypti* and (B) *Ae*. *albopictus*. A no-template control (NTC) was included. These results constitute part of the data included in Table 2.
(PNG)

S1 Table Summary of *Ae. aegypti* LAMP and TaqMan results. (DOCX)

S2 Table Summary of *Ae. albopictus* LAMP and TaqMan results. (DOCX)

S3 Table **Detection of *Aedes aegypti* DNA in 1 mL water aliquots removed from (a) water in which eggs had been hatched and larvae reared in (Experiments 1 and 2) and (b) water in which larvae had been transferred into 2 h after hatching (Experiment 3)**.
(DOCX)
