# Supplementary figures and images for: A LAMP-based colorimetric assay to expedite field surveillance of the invasive mosquito species Aedes aegypti and Aedes albopictus
Source: PLoS Negl Trop Dis. 2020 Mar 4;14(3):e0008130. doi: 10.1371/journal.pntd.0008130 (PMC7055815; doi:10.1371/journal.pntd.0008130)

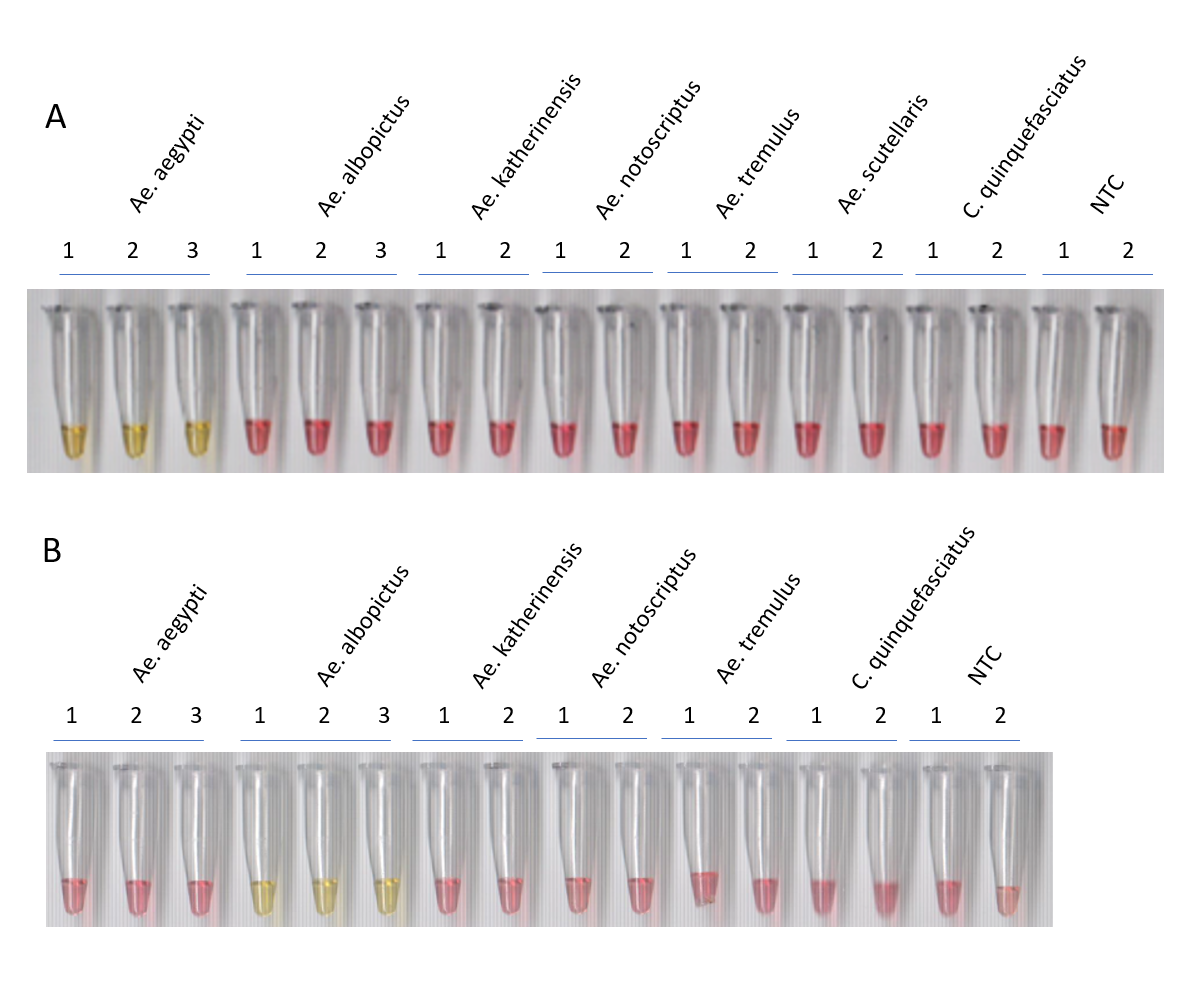

Supplement: S1 Fig — As indicated, mosquito species were tested in duplicate or triplicate with LAMP reactions to detect (A) Ae. aegypti and (B) Ae. albopictus. A no-template control (NTC) was included. These results constitute part of the data included in Table 2. (PNG) [file pntd.0008130.s001.png]
