# Supplementary material for: A LAMP-based colorimetric assay to expedite field surveillance of the invasive mosquito species Aedes aegypti and Aedes albopictus
Source: PLoS Negl Trop Dis. 2020 Mar 4;14(3):e0008130. doi: 10.1371/journal.pntd.0008130 (PMC7055815; doi:10.1371/journal.pntd.0008130)
Supplement: S1 Table — (DOCX) [file pntd.0008130.s002.docx]

**S1 Table. Summary of *Ae. aegypti* LAMP and TaqMan results.**

|  | Mosquito 1 | |  | Mosquito 2 | |
| --- | --- | --- | --- | --- | --- |
| Dilution (fold) | LAMP assay | C_t_ |  | LAMP assay | C_t_ |
| 10^2^ | + | 16.45 |  | + | 16.52 |
| “ | + | 16.03 |  | + | 16.47 |
| 10^3^ | + | 18.94 |  | + | 20.11 |
| “ | + | 18.72 |  | + | 20.28 |
| 10^4^ | + | 24.18 |  | + | 25.26 |
| “ | + | 23.83 |  | + | 25.15 |
| 10^5^ | + | 27.98 |  | + | 29.21 |
| “ | + | 28.01 |  | + | 29.17 |
| 10^6^ | + | 32.65 |  | - | 33.72 |
| “ | + | 32.44 |  | + | 33.77 |
| 10^7^ | + | 36.65 |  | - | 39.25 |
| “ | - | 36.85 |  | - | 38.08 |
| 10^8^ | - | ≥ 39 |  | - | ≥ 39 |
| “ | - | ≥ 39 |  | - | ≥ 39 |
| 10^9^ | - | ≥ 39 |  | - | ND |
| “ | - | ≥ 39 |  | - | ≥ 39 |
| 10^10^ | - | ≥ 39 |  | - | ≥ 39 |
| “ | - | ≥ 39 |  | - | ≥ 39 |
| NTC | - | ≥ 39 |  |  |  |
| “ | - | ≥ 39 |  |  |  |

^a^A sample was detected if the cycle threshold (C_t_) value was < 39.0 cycles. Ct values ≥ 39.0 were considered to be not detected.
