# Supplementary material for: A LAMP-based colorimetric assay to expedite field surveillance of the invasive mosquito species Aedes aegypti and Aedes albopictus
Source: PLoS Negl Trop Dis. 2020 Mar 4;14(3):e0008130. doi: 10.1371/journal.pntd.0008130 (PMC7055815; doi:10.1371/journal.pntd.0008130)
Supplement: S2 Table — (DOCX) [file pntd.0008130.s003.docx]

**S2 Table. Summary of *Ae. albopictus* LAMP and TaqMan results.**

|  | Mosquito 1 | |  | Mosquito 2 | |
| --- | --- | --- | --- | --- | --- |
| Dilution (fold) | LAMP | C_t_ |  | LAMP | C_t_ |
| 10^2^ | + | 21.05 |  | + | 21.17 |
| “ | + | 21.15 |  | + | 21.05 |
| 10^3^ | + | 24.93 |  | + | 24.5 |
| “ | + | 25.3 |  | + | 24.27 |
| 10^4^ | + | 31.36 |  | + | 30.69 |
| “ | + | 31.41 |  | + | 30.13 |
| 10^5^ | + | 34.93 |  | - | 35.23 |
| “ | - | 34.8 |  | - | 35.76 |
| 10^6^ | - | ≥ 39 |  | - | 39.07 |
| “ | - | ≥ 39 |  | - | 37.85 |
| 10^7^ | - | ≥ 39 |  | - | ≥ 39 |
| “ | - | ≥ 39 |  | - | ≥ 39 |
| NTC | - | ≥ 39 |  |  |  |
| “ | - | ≥ 39 |  |  |  |

^a^A sample was detected if the cycle threshold (C_t_) value was < 39.0 cycles. Ct values ≥ 39.0 were considered to be not detected.
