# Supplementary material for: A LAMP-based colorimetric assay to expedite field surveillance of the invasive mosquito species Aedes aegypti and Aedes albopictus
Source: PLoS Negl Trop Dis. 2020 Mar 4;14(3):e0008130. doi: 10.1371/journal.pntd.0008130 (PMC7055815; doi:10.1371/journal.pntd.0008130)
Supplement: S3 Table — Detection of Aedes aegypti DNA in 1 mL water aliquots removed from (a) water in which eggs had been hatched and larvae reared in (Experiments 1 and 2) and (b) water in which larvae had been transferred into 2 h after hatching (Experiment 3). (DOCX) [file pntd.0008130.s004.docx]

**S3 Table. Detection of *Aedes aegypti* DNA in 1 mL water aliquots removed from (a) water in which eggs had been hatched and larvae reared in (Experiments 1 and 2) and (b) water in which larvae had been transferred into 2 h after hatching (Experiment 3).**

|  |  |  |  |  | *Ae. aegypti* LAMP assay result | | *Ae. aegypti* TaqMan RT-PCR result | | | | |
| --- | --- | --- | --- | --- | --- | --- | --- | --- | --- | --- | --- |
| Sample type | Day post hatching | Number of *Ae. aegypti* larvae | Number samples tested |  | Number (%) positive | | Number (%) positive | | C_t_ score (Mean ± SD)^a^ | | |
| Hatching water  (Experiment 1) | 1 | 0 | 3 |  | 0 | (0) | 0 | (0) | ≥ 39 | | |
|  |  | 3 - 9 | 3 |  | 3 | (100) | 3 | (100) | 25.1 | ± | 3.2 |
|  |  | 91-111 | 3 |  | 3 | (100) | 3 | (100) | 21.5 | ± | 1.1 |
| Hatching water (Experiment 2) | 1 | 0 | 2 |  | 0 | (0) | Not tested | | | | |
|  |  | 1-4 | 10 |  | 2 | (20) | Not tested | | | | |
|  | 2 | 0 | 2 |  | 0 | (0) | Not tested | | | | |
|  | 2 | 1-4 | 10 |  | 2 | (20) | Not tested | | | | |
| Rearing water  (Experiment 3) | 1 | 0 | 2 |  | 0 | (0) | 0 | (0) | ≥ 39 | | |
|  |  | 1 | 5 |  | 2 | (40) | 3 | (60) | 33.4 | ± | 0.2 |
|  |  | 5 | 5 |  | 2 | (40) | 3 | (60) | 31.6 | ± | 0.3 |
|  | 2 | 0 | 2 |  | 0 | (0) | 0 | (0) | ≥ 39 | | |
|  |  | 1 | 5 |  | 1 | (20) | 3 | (60) | 33.4 | ± | 0.6 |
|  |  | 5 | 5 |  | 0 | (0) | 3 | (60) | 33.0 | ± | 0.5 |
|  | 3 | 0 | 2 |  | 0 | (0) | 0 | (0) | ≥ 39 | | |
|  |  | 1 | 5 |  | 0 | (0) | 1 | (20) | 34.4 | | |
|  |  | 5 | 5 |  | 0 | (0) | 2 | (40) | 36.6 | ± | 0.0 |
|  | 7 | 0 | 2 |  | 0 | (0) | 0 | (0) | ≥ 39 | | |
|  |  | 1 | 5 |  | 0 | (0) | 0 | (0) | ≥ 39 | | |
|  |  | 5 | 5 |  | 0 | (0) | 3 | (60) | 35.4 | ± | 3.7 |

^a^A sample was detected if the cycle threshold (C_t_) value was < 39.0 cycles. Ct values ≥ 39.0 were considered to be not detected.
